# Immunomodulatory effects of a multi-component pharmacological intervention on diabetic peripheral neuropathy in type 2 diabetic rats: An exploratory study

**DOI:** 10.1371/journal.pone.0350984

**Published:** 2026-06-04

**Authors:** Lu Zhang, Si Wang, Jie Lei, Lingrui Zeng, Ailin Lu, Yongqing Wu, Yuan Shi, Jing Yang, Mengrui Yuan, Hongyi Liu

**Affiliations:** 1 Cancer Precision Medicine Center, Tianjin Cancer Hospital Airport Hospital, Tianjin, China; 2 The Second Clinical Medical College, Yunnan University of Traditional Chinese Medicine, Yunnan, China; 3 The First Clinical Medical College, Yunnan University of Traditional Chinese Medicine, Yunnan, China; 4 Scientific Research Department, Yunnan Provincial Hospital of Traditional Chinese Medicine, Yunnan, China; Università degli Studi della Campania, ITALY

## Abstract

**Background:**

Diabetic peripheral neuropathy (DPN) is a common complication of type 2 diabetes mellitus (T2DM) and is closely linked to immune and inflammatory dysregulation. Multi-component pharmacological interventions have been explored as complementary approaches for metabolic and immune modulation; however, their effects on DPN and related mechanisms remain incompletely understood.

**Methods:**

A rat model of T2DM-associated peripheral neuropathy was established, and a multi-component pharmacological intervention (MPCI) was administered for 8 weeks. Peripheral nerve dysfunction was evaluated by motor and sensory nerve conduction velocities (MNCV and SNCV), behavioral outcomes, and histological/ultrastructural assessments. In parallel, spleen tissues were collected for transcriptomic profiling. RNA sequencing was performed to identify differentially expressed genes and immune-related pathways, and representative molecules involved in inflammatory regulation were further validated using western blotting and quantitative real-time PCR in sciatic nerve tissue.

**Results:**

MPCI administration significantly ameliorated peripheral nerve dysfunction in T2DM rats, as evidenced by improved nerve conduction velocities and pathological features. Transcriptomic analysis of spleen tissue revealed that MPCI was associated with broad remodeling of diabetes-related immune and inflammatory gene programs. In parallel, sciatic nerve analyses showed attenuation of NF-κB/c-Jun–associated inflammatory signaling and modulation of inhibitory regulators at both the protein and mRNA levels.

**Conclusion:**

These findings indicate that MPCI improves T2DM-associated DPN and is associated with splenic immune remodeling and attenuation of peripheral nerve inflammatory signaling, providing exploratory evidence for associations between splenic immune transcriptomic remodeling and peripheral nerve inflammatory signaling.

## 1. Introduction

Type 2 diabetes mellitus (T2DM) is a complex metabolic disorder characterized by insulin resistance and chronic hyperglycemia. Beyond metabolic abnormalities, T2DM is increasingly recognized as an immune-metabolic disease accompanied by persistent low-grade inflammation and systemic immune dysregulation [1]. Aberrant activation of immune and inflammatory pathways contributes not only to impaired glucose metabolism but also to diabetic complications, including diabetic peripheral neuropathy (DPN) [2], which severely affects quality of life.

Immune-mediated inflammation plays an important role in the pathogenesis of DPN [3]. Peripheral nerves are susceptible to inflammatory injury, and sustained activation of pro-inflammatory signaling pathways has been implicated in nerve dysfunction and structural damage in diabetes [4,5]. However, upstream immune regulatory mechanisms that sustain neuroinflammation in T2DM remain incompletely understood. In particular, whether immune-organ transcriptional remodeling contributes to the inflammatory context associated with DPN has not been fully clarified.

The spleen is the largest secondary lymphoid organ and a central hub for coordinating innate and adaptive immune responses. Beyond its classical immunological functions, the spleen is increasingly recognized as a regulator of systemic inflammatory tone and immune-metabolic crosstalk [6–8]. Dysregulated splenic immune responses have been linked to chronic inflammatory conditions and metabolic disorders [9]. Splenic immune activation may be associated with systemic inflammatory changes in T2DM, but its relationship with peripheral nerve inflammatory injury remains unclear.

Traditional medicine-derived multi-component pharmacological intervention (MPCI) often exhibits pleiotropic, system-level pharmacological actions and may be suitable for multifactorial diseases such as T2DM [10–12]. MPCI is a traditional multi-herbal formula derived from a clinic-based prescription and prepared as a fixed-batch extract. Although its chemical profile has been characterized by UPLC-Q-TOF-MS, its immunomodulatory effects in T2DM-associated DPN remain incompletely understood. Moreover, previous studies of multi-component interventions in DPN have mainly focused on glucose metabolism, oxidative stress, or local nerve protection, whereas integration of immune-organ transcriptomics with peripheral nerve inflammatory signaling analysis remains limited.

Nuclear factor kappa B (NF-κB) and activator protein-1 (AP-1), of which c-Jun is a major component, are central transcriptional regulators of inflammatory gene expression and immune activation [13,14]. Negative regulators such as IκBα and A20 (Tnfaip3) are essential for maintaining immune homeostasis by restraining excessive inflammatory signaling [15,16]. Whether MPCI modulates the NF-κB/c-Jun axis in the context of T2DM-associated immune dysregulation and neuroinflammation has not been determined.

In this exploratory study, we used the HFD/STZ-induced T2DM rat model to evaluate the effects of MPCI on DPN-related functional, metabolic, and pathological outcomes. We also performed spleen RNA-seq to explore MPCI-associated transcriptomic changes and examined selected NF-κB/c-Jun–related inflammatory markers in sciatic nerve tissue. This study aimed to describe potential associations between splenic immune transcriptomic remodeling and peripheral nerve inflammatory signaling, rather than to establish a causal spleen–nerve mechanism.

## 2. Methods

### 2.1 Animals, diabetes induction, and treatment

Male Wistar rats (180–220 g) were housed under standard laboratory conditions with ad libitum access to food and water. After one week of acclimatization, rats were randomized to a control group (standard chow) or a model group fed a high-fat diet (HFD; 67% basal diet, 10% lard, 20% sucrose, 2.5% cholesterol, 0.5% sodium cholate). After 8 weeks of HFD feeding, T2DM was induced by intraperitoneal streptozotocin (STZ, 35 mg/kg) for 3 consecutive days (citrate buffer, pH 4.5). Rats with fasting blood glucose (FBG) > 11.1 mmol/L were considered diabetic. After confirmation of diabetes, rats were randomized into the Model (vehicle), MPCI-L (7.64 g/kg/day), MPCI-M (15.28 g/kg/day), MPCI-H (30.56 g/kg/day) and Mecobalamin (189 mg/kg/day) groups. MPCI and mecobalamin were administered by oral gavage once daily for 8 weeks, while control and model rats received an equivalent volume of vehicle. Mecobalamin was included as a positive control for phenotypic evaluation of diabetic peripheral neuropathy. Randomization was performed using a computer-generated random number sequence. Outcome assessments and data analyses were conducted blinded to group allocation whenever applicable. All animal experimental procedures were approved by the Ethical Committee of Yunnan University of Traditional Chinese Medicine (approval No. R-062023G084) and were conducted in accordance with relevant guidelines and regulations.

Humane endpoints were predefined for all animals. Rats were monitored at least once daily throughout the study and twice daily during STZ administration and for 1 week thereafter. Animals were euthanized if they showed >20% body weight loss, inability to access food or water, severe lethargy or persistent recumbency, marked dehydration, labored breathing, persistent hypothermia, or other moribund signs; severe hyperglycemia with rapid deterioration in general condition was also considered a humane endpoint. A total of 70 rats were used, of which 10 served as the Control group without diabetes induction and the remaining 60 underwent HFD/STZ induction. During model induction and observation, 3 rats died spontaneously, 6 were euthanized after reaching humane endpoints, and 1 failed to meet the diabetic criterion and was excluded; the remaining rats were included in subsequent group allocation. Because the mechanistic focus of this study was MPCI-associated immune transcriptional remodeling and NF-κB/c-Jun signaling validation, transcriptomic sequencing, western blotting, and qPCR analyses were performed only in the Control, Model, and MPCI-treated groups, but not in the Mecobalamin group. Euthanasia was performed by cervical dislocation under deep isoflurane anesthesia, and death was confirmed by cessation of respiration and heartbeat. All animal procedures were performed by trained personnel with institutional training in animal handling, welfare monitoring, recognition of pain, distress, and euthanasia.

### 2.2 Sample collection and biochemical analysis

After overnight fasting, blood was collected from the inner canthus under light anesthesia, and serum was separated by centrifugation. FBG was measured using a glucose meter (Roche, Switzerland). Fasting serum insulin (FINS) was determined by radioimmunoassay kits (Beijing Sino-UK Institute of Biological Technology, China). Insulin resistance was assessed using Homeostasis Model Assessment of Insulin Resistance (HOMA-IR), calculated as [FBG (mmol/L) × FINS (μU/mL)] / 22.5. Insulin sensitivity was evaluated using HOMA-I, calculated as ln[1 / (FBG × FINS)] [12].

### 2.3 Assessment of peripheral nerve function

Rats were anesthetized with isoflurane, and motor nerve conduction velocity (MNCV) and sensory nerve conduction velocity (SNCV) [17] were measured using a BL-420N biological signal acquisition and processing system. The sciatic nerve was stimulated at proximal and distal sites, compound action potentials were recorded, and conduction velocity was calculated as the distance between the two stimulation sites divided by the difference in onset latency between proximal and distal stimulations.

### 2.4 Organ indices and histopathology

Spleen and kidney indices were calculated as organ weight/body weight (×100%). Tissues were fixed in 4% paraformaldehyde, paraffin-embedded, sectioned, and stained with hematoxylin and eosin (H&E) for histopathological examination. For transmission electron microscopy (TEM), samples were fixed in glutaraldehyde, post-fixed, dehydrated, embedded, sectioned, and examined under an electron microscope to assess ultrastructural alterations.

### 2.5 Western blotting

Sciatic nerve tissues were lysed using RIPA buffer (R0010; Solarbio) supplemented with PMSF (P0100; Solarbio). Protein concentrations were measured using a BCA assay, and equal amounts of protein were separated by SDS–PAGE and transferred to PVDF membranes (Millipore). Membranes were blocked with 5% non-fat milk in TBST and incubated with primary antibodies against phosphorylated c-Jun (p-cJun; ab32385; Abcam), phosphorylated NF-κB p65 (ab76302; Abcam), IκBα (ab32518; Abcam), and A20/Tnfaip3 (5630S; Cell Signaling Technology), followed by HRP-conjugated secondary antibodies. Chemiluminescent signals were developed using Super ECL Plus (S6009; US EVERBRIGHT INC.) and captured using a ChemiDoc XRS+ imaging system (Bio-Rad). Band intensities were quantified using ImageJ and normalized to the corresponding loading controls (tubulin, GAPDH, or β-actin, as applicable).

### 2.6 Quantitative real-time PCR

Total RNA was extracted from sciatic nerve tissues using TriQuick Reagent (R1100; Solarbio, China) and reverse-transcribed using a commercial cDNA synthesis kit (Servicebio, China) according to the manufacturers’ instructions. Quantitative real-time PCR was performed using SYBR Green qPCR master mix (Servicebio, China) on a Q2000B real-time PCR system (Hangzhou Longgene Scientific Instruments, China). Relative mRNA levels of Jun, Rela, Nfkbia, and Tnfaip3 were calculated using the 2^ − ΔΔCt method and normalized to Gapdh. Primer sequences are provided in S1 Table.

### 2.7 RNA sequencing and bioinformatics analysis

Total RNA from spleen tissues was used for transcriptome sequencing, and RNA integrity was assessed using an Agilent Bioanalyzer 2100. Libraries were constructed using the Fast RNA-seq Lib Prep Kit V2 (Cat. No. RK20306) following the manufacturer’s instructions. Briefly, mRNA was purified using poly-T oligo–attached magnetic beads, fragmented, and reverse-transcribed with random hexamer primers to generate first-strand cDNA, followed by second-strand synthesis. Libraries were then prepared through end repair, A-tailing, adapter ligation, size selection, PCR amplification, and purification. Libraries were quantified by Qubit and real-time PCR and assessed for size distribution by Bioanalyzer prior to sequencing on an Illumina NovaSeq X Plus platform.

Raw reads were quality-filtered using fastp to remove adapter-contaminated, poly-N, and low-quality reads, and Q20/Q30 and GC content were calculated. Clean reads were aligned to the reference genome (Rattus norvegicus mRatBN7.2) using RNA STAR [18], and gene-level counts were generated using featureCounts [19]. Differentially expressed genes (DEGs) were identified using DESeq2 [20] with Benjamini-Hochberg multiple-testing correction; genes with FDR < 0.05 and |log2FC| ≥ 1 were considered differentially expressed. Gene Ontology (GO) enrichment analysis and Gene Set Enrichment Analysis (GSEA) [21] were performed to characterize diabetes- and MPCI-associated transcriptional changes.

### 2.8 MPCI preparation and chemical characterization

MPCI was derived from a traditional multi-herbal prescription. Each dose unit consisted of *Rhei Radix et Rhizoma* (6 g), *Coptidis Rhizoma* (15 g), *Curcumae Longae Rhizoma* (15 g), *Bombyx Batryticatus* (6 g), *Cicadae Periostracum* (6 g), *Aurantii Fructus Immaturus* (10 g), processed *Pinelliae Rhizoma* (10 g), *Bupleuri Radix* (15 g), *Paeoniae Radix Alba* (20 g), *Scutellariae Radix* (15 g), *Zingiberis Rhizoma* (5 g), *Eupatorii Herba* (20 g), *Toddalia asiatica* (30 g), *Gaultheriae Yunnanensis Herba* (30 g), and *Resina Draconis* (6 g). All herbal materials were purchased from the Yunnan Provincial Hospital of Traditional Chinese Medicine and authenticated by qualified professionals before use. The herbal materials were mixed, soaked in 10 volumes of purified water for 30 min, and decocted twice for 30 min each. The two extracts were filtered, combined, and concentrated under reduced pressure to an appropriate volume to obtain a final preparation equivalent to 4 g/mL crude drug. To minimize batch-to-batch variation, all animal experiments in the present study were conducted using the same production batch of MPCI. Chemical profiling of the administered extract and post-dosing serum samples was performed using UPLC-Q-TOF-MS. Compound identification was based on accurate mass measurements, MS/MS fragmentation patterns, and database/literature matching. Representative chromatograms and compound annotation data are provided in the Supplementary Materials.

### 2.9 Statistical analysis

Statistical analyses were performed using GraphPad Prism 10. Data are presented as mean ± SD. Differences among groups were analyzed by one-way ANOVA followed by Tukey’s multiple comparisons test. A two-sided p value < 0.05 was considered statistically significant. Sample sizes varied by assay: n = 8 per group for functional, biochemical, histological, and TEM analyses; n = 4 per group for RNA-seq; n = 3 per group for western blotting; and n = 5 per group for qPCR. Transcriptomic, western blotting, and qPCR analyses included only the Control, Model, and MPCI-treated groups, whereas the Mecobalamin group was included in phenotypic, biochemical, and histopathological assessments. Sample sizes are also indicated in the corresponding figure legends.

## 3. Results

### 3.1 Experimental design, body weight, and peripheral nerve function

**Fig 1:**
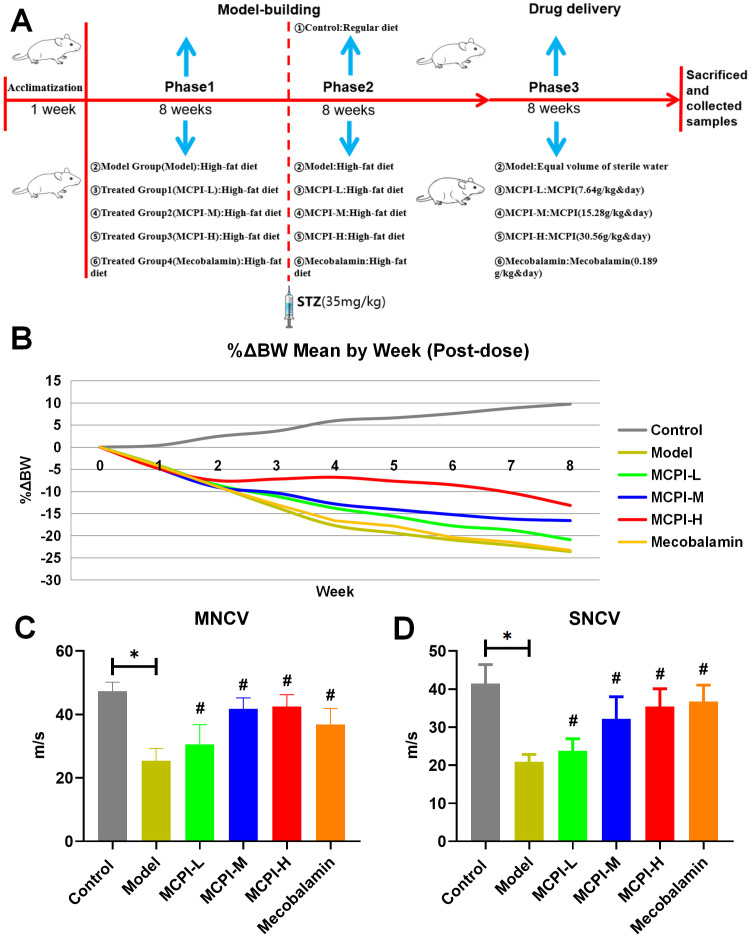
Experimental design and effects of MPCI on body weight and peripheral nerve function in HFD/STZ-induced T2DM rats. (A) Schematic overview of the experimental design. After a one-week acclimatization period, rats were fed either a regular diet (Control) or a high-fat diet (HFD). T2DM was induced by intraperitoneal injection of streptozotocin (STZ, 35 mg/kg). After model establishment, rats were treated with vehicle or MPCI at low (MPCI-L), medium (MPCI-M), high (MPCI-H) doses, or mecobalamin as a positive control for 8 weeks. Animals were then sacrificed and samples were collected for subsequent analyses. (B) Percentage change in body weight (%ΔBW) during the treatment period. (C) Motor nerve conduction velocity (MNCV) and (D) sensory nerve conduction velocity (SNCV) were measured to assess peripheral nerve function. Data are presented as mean ± SD (n = 8 per group). *P < 0.05 vs. Control; #P < 0.05 vs. Model.

After model establishment, rats received vehicle or MPCI at low, medium, or high doses for 8 weeks (Fig 1A). During the treatment period, diabetic rats showed a diabetes-associated body weight loss trajectory compared with controls, whereas MPCI partially attenuated diabetes-associated body weight loss, with the effect being most evident in the high-dose group (Fig 1B).

Peripheral nerve function was assessed by MNCV and SNCV. Compared with control rats, diabetic rats exhibited significant reductions in both MNCV and SNCV, indicating impaired peripheral nerve function consistent with diabetic peripheral neuropathy (Fig 1C-1D). MPCI treatment markedly increased MNCV and SNCV relative to the Model group, with medium- and high-dose MPCI restoring nerve conduction velocities toward control levels (Fig 1C-1D). These findings suggest that MPCI ameliorates diabetes-associated peripheral nerve dysfunction.

### 3.2 MPCI attenuates metabolic disturbance and insulin resistance

**Fig 2:**
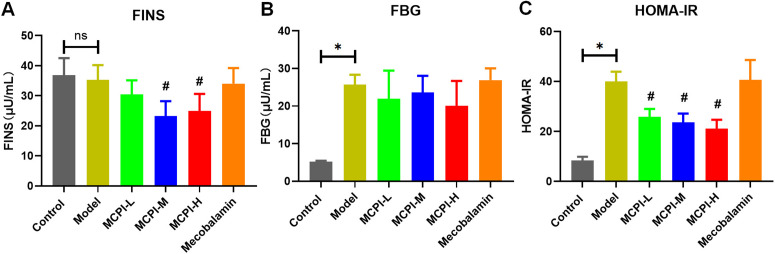
Effects of MPCI on glucose metabolism and insulin resistance in HFD/STZ-induced T2DM rats. (A) Fasting insulin (FINS). (B) Fasting blood glucose (FBG). (C) Homeostasis model assessment of insulin resistance (HOMA-IR). Data are presented as mean ± SD (n = 8 per group). *P < 0.05 vs. Control; #P < 0.05 vs. Model.

To evaluate metabolic effects of MPCI, FBG, FINS, and HOMA-IR were assessed. Diabetic rats exhibited markedly elevated FBG and HOMA-IR compared with controls, whereas FINS levels were not significantly different between the Model and Control groups, consistent with an insulin resistance–dominant metabolic phenotype (Figs 2A–2C). MPCI treatment reduced HOMA-IR relative to the Model group across the MPCI-treated groups, with medium- and high-dose MPCI also significantly reducing FINS levels. FBG tended to decrease after MPCI treatment, particularly in the high-dose group, although the reduction was not statistically significant in the present dataset. These results indicate that MPCI attenuates insulin resistance and partially improves metabolic disturbance in HFD/STZ-induced T2DM rats.

### 3.3 MPCI alleviates diabetes-associated histopathological and ultrastructural damage in the sciatic nerve

**Fig 3:**
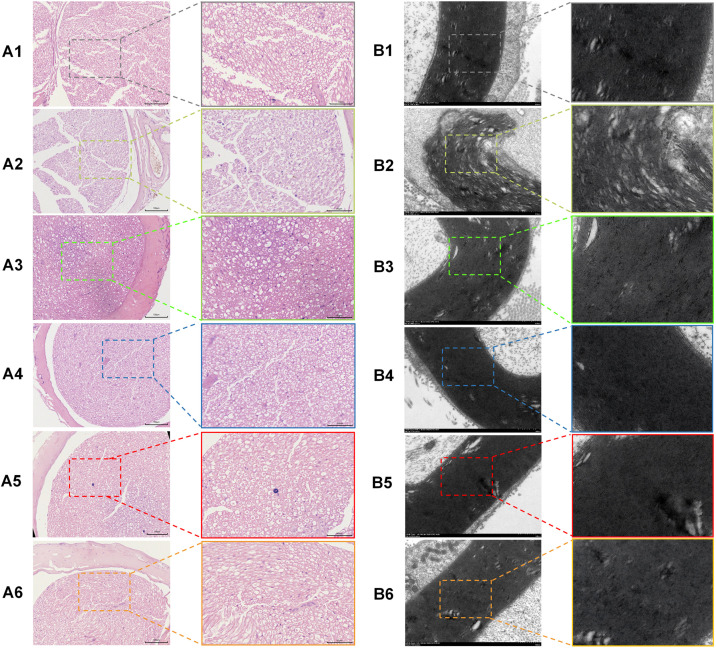
MPCI alleviates diabetes-associated histopathological and ultrastructural damage in the sciatic nerve. (A1–A6) Representative H&E-stained sections of sciatic nerve from Control (A1), Model (A2), MPCI-L (A3), MPCI-M (A4), MPCI-H (A5), and Mecobalamin (A6) groups. Compared with the Control group, diabetic model rats exhibited disorganized nerve fibers and prominent vacuolation-like changes. MPCI treatment partially alleviated these pathological alterations, as reflected by relatively improved fiber organization and reduced vacuolation-like changes, particularly in the medium- and high-dose groups. Mecobalamin treatment also showed a protective effect on sciatic nerve morphology. (B1–B6) Representative TEM images of sciatic nerve ultrastructure from Control (B1), Model (B2), MPCI-L (B3), MPCI-M (B4), MPCI-H (B5), and Mecobalamin (B6) groups. Diabetic model rats showed marked myelin lamellar disorganization, loosening/splitting, and whorl-like profiles. MPCI treatment partially improved myelin ultrastructural integrity, with relatively preserved myelin organization observed in the treated groups, particularly in the medium- and high-dose groups. Mecobalamin treatment also exerted a protective effect on sciatic nerve ultrastructure. Scale bars: 100 μm for H&E images; 500 nm for TEM images. Representative images are shown from n = 8 animals per group.

Histopathological examination of sciatic nerve tissue was performed to evaluate diabetes-associated peripheral nerve injury and the effects of MPCI. H&E staining showed relatively intact and regularly arranged nerve fibers in the control group, whereas diabetic model rats exhibited disorganized nerve fibers and prominent vacuolation-like changes (Fig 3 A1–A6). MPCI treatment partially alleviated these pathological alterations, as reflected by relatively improved fiber organization and reduced vacuolation-like changes, particularly in the medium- and high-dose groups. The mecobalamin-treated group also showed partial improvement in sciatic nerve morphology.

Consistently, TEM demonstrated marked ultrastructural abnormalities in the sciatic nerve of diabetic rats, characterized by myelin lamellar disorganization with loosening/splitting and whorl-like profiles (Fig 3 B1-B6). MPCI treatment partially improved myelin ultrastructural integrity, with relatively preserved myelin organization observed in the treated groups, particularly in the medium- and high-dose groups. Mecobalamin treatment also showed a protective effect on sciatic nerve ultrastructure. Together, these morphological findings provide supportive evidence that MPCI attenuates diabetes-associated sciatic nerve damage.

### 3.4 MPCI modulates diabetes-associated splenic transcriptomic alterations

**Fig 4:**
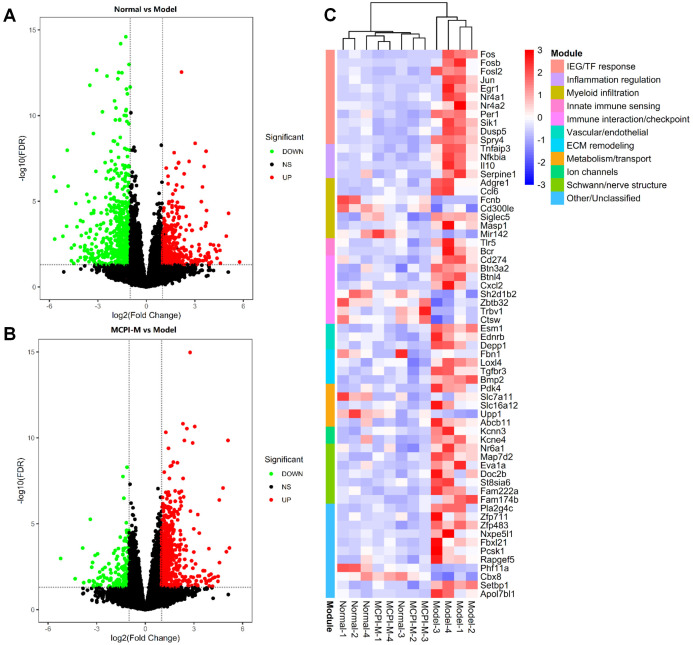
Splenic transcriptomic remodeling associated with MPCI treatment in HFD/STZ-induced T2DM rats. (A) Volcano plot of DEGs between Control and Model groups in spleen tissue. (B) Volcano plot of DEGs between Model and MPCI-M groups. (C) Heatmap of shared DEGs identified across comparisons, highlighting diabetes-associated genes modulated by MPCI treatment. RNA-seq was performed using n = 4 independent spleen samples per group.

Given the central role of the spleen in systemic immune regulation, RNA-seq was performed to characterize diabetes-associated transcriptional changes in spleen tissue and their modulation by MPCI. Volcano plot analysis revealed extensive DEGs between the Control and Model groups, indicating pronounced diabetes-associated transcriptomic dysregulation in the spleen (Fig 4A). In the comparison between Model and MPCI-M groups, MPCI markedly altered gene expression patterns in diabetic rats (Fig 4B).

GSEA of GO biological process gene sets revealed distinct enrichment patterns across the two comparisons (S1 and S2 Figs). In the Control versus Model comparison, enriched gene sets were mainly related to broad diabetes-associated transcriptional alterations, including chromosome segregation, cell division, and developmental or organ-related processes (S1 Fig). In contrast, the Model versus MPCI-M comparison highlighted MPCI-associated modulation of immune- and inflammation-related biological processes, including inflammatory regulation, innate immune responses, and myeloid cell–associated signatures (S2 Fig). These findings suggest that MPCI treatment reshaped splenic transcriptional programs in diabetic rats, with prominent effects on immune- and inflammation-related pathways. Heatmap analysis of shared DEGs across the Control vs Model and Model vs MPCI-M comparisons further demonstrated that MPCI shifted a subset of diabetes-associated splenic gene expression changes away from the diabetic state (Fig 4C). Functional module annotation highlighted coordinated regulation of immediate early gene/transcription factor responses (e.g., Fos/Jun/Egr1) and inflammatory regulators (e.g., Nfkbia and Tnfaip3), providing transcriptomic context for subsequent pathway validation in peripheral nerve tissue.

### 3.5 MPCI attenuates NF-κB/c-Jun–associated inflammatory signaling and modulates endogenous feedback regulators in the sciatic nerve

**Fig 5:**
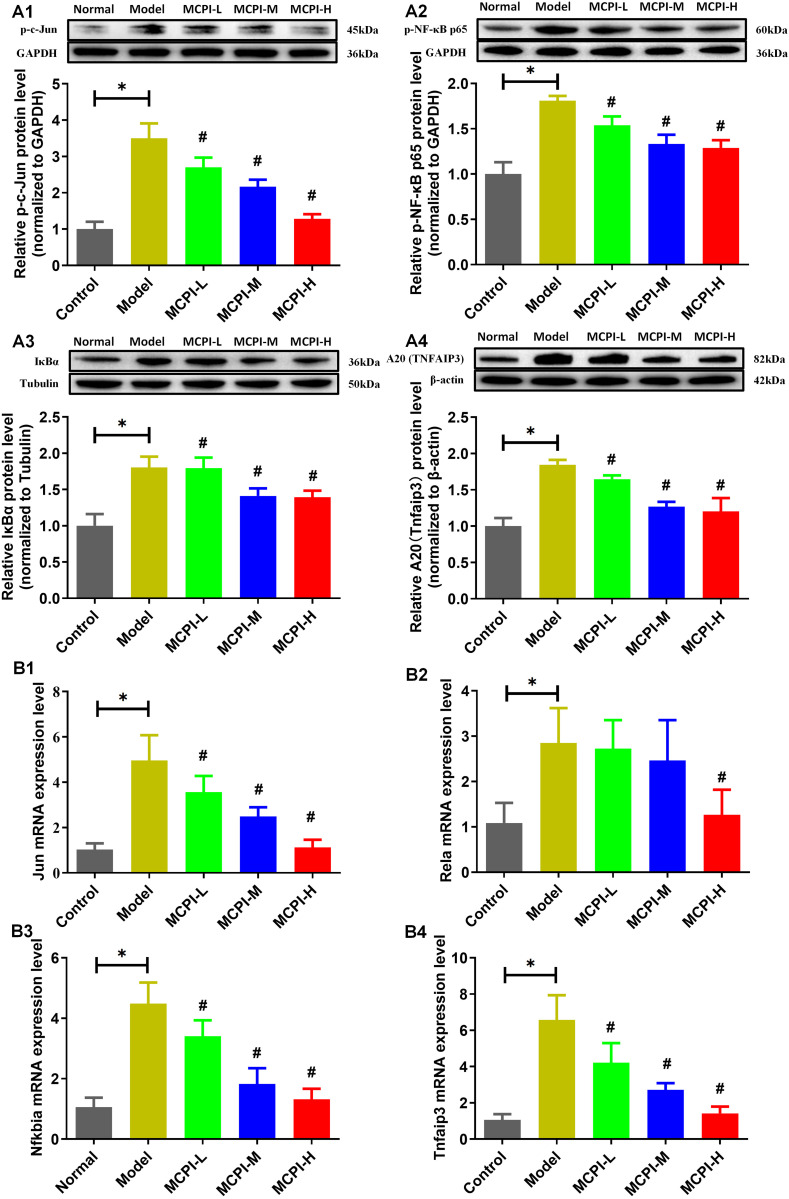
MPCI attenuates NF-κB/c-Jun–associated inflammatory signaling and modulates endogenous feedback regulators in sciatic nerve tissue. (A1–A4) Representative Western blots and quantification of p-c-Jun, p-NF-κB p65 (Ser536), IκBα, and A20 (TNFAIP3) in sciatic nerve tissues from Control, Model, MPCI-L, MPCI-M, and MPCI-H groups. Protein levels were normalized to GAPDH, tubulin, or β-actin as indicated. (B1–B4) qPCR analysis of Jun, Rela, Nfkbia, and Tnfaip3 mRNA expression in sciatic nerve tissues. Data are presented as mean ± SD. Western blotting was performed using n = 3 independent biological replicates per group, and qPCR was performed using n = 5 independent biological replicates per group. *P < 0.05 vs. Control; #P < 0.05 vs. Model. Increased IκBα and A20 expression in the Model group may reflect compensatory negative-feedback activation, and MPCI-associated changes should be interpreted as feedback modulation rather than simple restoration toward control levels.

To explore molecular changes in peripheral nerve tissue associated with MPCI-mediated neuroprotection, key components of inflammatory signaling were examined in sciatic nerves. Western blotting showed that phosphorylation of NF-κB p65 (Ser536) and c-Jun was significantly increased in diabetic rats, indicating activation of pro-inflammatory signaling in the sciatic nerve (Fig 5 A1–A2). MPCI treatment markedly reduced p-c-Jun and p-NF-κB p65 levels, with more evident effects observed in the medium- and high-dose groups. Notably, the inhibitory regulatory proteins IκBα and A20 were also increased in the Model group compared with the Control group (Fig 5 A3–A4). Rather than indicating reduced inhibitory regulation, this pattern may reflect compensatory activation of endogenous negative-feedback mechanisms in response to sustained inflammatory signaling. MPCI treatment decreased the elevated IκBα and A20 protein levels relative to the Model group, suggesting modulation of inflammatory feedback regulation rather than simple restoration toward control levels.

Consistent with the protein-level findings, qPCR analysis indicated that diabetes increased the mRNA expression of Jun, Rela, Nfkbia, and Tnfaip3 in sciatic nerve tissue (Fig 5 B1–B4). MPCI treatment reduced these inflammatory and regulatory transcripts to varying degrees, supporting its association with attenuation of NF-κB/c-Jun–related inflammatory activation and modulation of endogenous feedback responses in peripheral nerve tissue.

### 3.6 Chemical characterization of MPCI and serum-exposed constituents

To improve the interpretability and reproducibility of MPCI, the administered extract was chemically profiled by UPLC-Q-TOF-MS in both positive and negative ion modes, and 111 constituents were putatively identified based on accurate mass and MS/MS fragmentation information with database/literature matching (S2 Table). Following oral administration, serum profiling detected 158 MPCI-related compounds, including 90 prototype constituents and 68 metabolites, supporting systemic exposure in vivo (S3 and S4 Tables).

### 3.7 Effects of MPCI on organ indices

**Fig 6:**
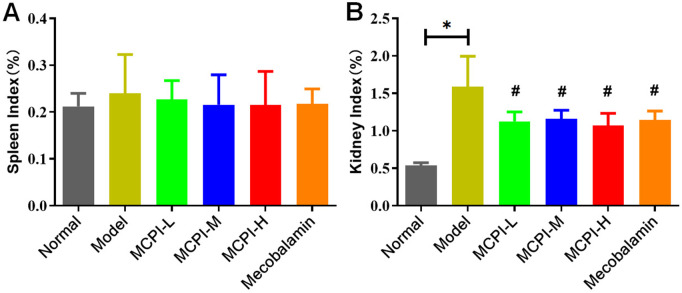
Effects of MPCI on spleen and kidney indices in HFD/STZ-induced T2DM rats. (A) Spleen index (%). (B) Kidney index (%). Data are presented as mean ± SD (n = 8 per group). *P < 0.05 vs. Control; #P < 0.05 vs. Model.

Spleen and kidney indices were calculated as organ weight/body weight × 100%. As shown in Fig 6A, the spleen index did not differ markedly among groups, with no significant changes observed in diabetic rats or following MPCI treatment. In contrast, the kidney index was significantly increased in the Model group compared with the Normal group (Fig 6B), suggesting diabetes-associated renal hypertrophic change. MPCI treatment at low, medium, and high doses significantly reduced the kidney index relative to the Model group, suggesting attenuation of this diabetes-associated morphometric alteration.

## 4. Discussion

DPN is a common and debilitating complication of T2DM, resulting from the combined effects of metabolic stress and chronic low-grade inflammation [22–24]. Increasing evidence suggests that systemic immune dysregulation, rather than hyperglycemia alone, plays a critical role in creating a pro-inflammatory milieu that predisposes peripheral nerves to functional impairment and structural damage [25–27]. Consistent with this view, multiple mechanistic and translational studies have highlighted persistent activation of inflammatory networks, including NF-κB–linked programs, as a core driver of peripheral nerve vulnerability and damage in diabetic neuropathy [28–30]. In this exploratory study, using the HFD/STZ-induced T2DM rat model, we found that MPCI improved peripheral nerve function and attenuated sciatic nerve injury. These effects were accompanied by splenic transcriptomic changes and reduced inflammatory signaling in peripheral nerve tissue.

Notably, FINS levels in the Model group were not significantly reduced relative to controls. This pattern may reflect the characteristics of the HFD/low-dose STZ protocol used in the present study, which is commonly used to generate a T2DM-like phenotype characterized by diet-induced insulin resistance with partial β-cell impairment rather than severe β-cell failure [31,32]. Under this condition, HFD-induced insulin resistance may elicit compensatory insulin secretion, thereby offsetting or masking the insulin-lowering effect of low-dose STZ [33]. Therefore, the coexistence of marked hyperglycemia and elevated HOMA-IR with relatively preserved fasting insulin levels suggests that insulin resistance, rather than severe insulin deficiency, was the predominant metabolic feature of this model [34].

The spleen is a key secondary lymphoid organ involved in systemic immune regulation [35,36]. A major exploratory component of this study was the spleen RNA-seq analysis. In diabetic rats, spleen transcriptomics showed broad diabetes-associated transcriptional alterations. After MPCI treatment, the Model versus MPCI-M comparison revealed changes in immune- and inflammation-related biological processes, including inflammatory regulation, innate immune responses, and myeloid cell–associated signatures. Heatmap analysis further suggested that MPCI shifted part of the diabetes-associated splenic gene expression pattern away from the diabetic state. These findings suggest that MPCI might be associated with remodeling of splenic immune-related transcriptional programs in diabetic rats. However, these transcriptomic results remain exploratory and do not establish a causal link between splenic immune remodeling and peripheral nerve protection.

We next examined NF-κB/c-Jun–associated inflammatory signaling in sciatic nerve tissue. NF-κB and c-Jun are key regulators of inflammatory responses and have been implicated in diabetic neuropathy-related nerve injury [37,38]. In diabetic rats, phosphorylation of NF-κB p65 and c-Jun was increased, together with increased expression of the inhibitory regulators IκBα and A20. This pattern may reflect activation of inflammatory signaling with compensatory feedback regulation. MPCI treatment reduced p-NF-κB p65 and p-c-Jun levels and modulated IκBα and A20 expression. These findings suggest that MPCI-associated improvement in peripheral nerve outcomes was accompanied by attenuation of NF-κB/c-Jun–related inflammatory signaling. However, these results should be interpreted as associated molecular changes rather than direct proof of a causal mechanism.

Mecobalamin was included as a positive control because it is commonly used as a neurotrophic agent in DPN studies [39,40]. MPCI produced functional and pathological improvements that were broadly comparable to those observed with mecobalamin. However, because mecobalamin was not included in the RNA-seq, western blotting, or qPCR analyses, this comparison should be interpreted only at the phenotypic level. UPLC-Q-TOF-MS analysis identified multiple MPCI-related prototype constituents and metabolites in circulation after oral administration, supporting systemic exposure in vivo. However, the specific contribution of individual compounds to the observed biological effects was not investigated [41–43].

This study has several limitations. First, it was exploratory in design. Although MPCI-related improvements in DPN outcomes were accompanied by splenic transcriptomic changes and reduced inflammatory signaling in sciatic nerve tissue, the present data do not establish a causal spleen–nerve mechanism. Second, the RNA-seq analysis was performed with a limited number of biological replicates (n = 4/group), which may restrict the robustness of transcriptomic interpretation. Third, immune-cell phenotyping, cytokine profiling, and functional immune validation experiments were not performed. Therefore, the proposed immunomodulatory interpretation should be considered preliminary. Fourth, kidney histopathology was not examined, so kidney index changes should be interpreted only as morphometric observations. Finally, although UPLC-Q-TOF-MS profiling supported systemic exposure to MPCI-related constituents, the contribution of individual compounds was not determined.

In summary, this study showed that MPCI alleviated DPN-related functional and pathological abnormalities in HFD/STZ-induced T2DM rats. These effects were associated with remodeling of diabetes-associated splenic immune transcriptional programs and attenuation of NF-κB/c-Jun–associated inflammatory signaling in sciatic nerve tissue. Rather than proving a direct spleen-to-nerve causal axis, the present findings provided preliminary evidence for associations between splenic immune transcriptomic remodeling and peripheral nerve inflammatory signaling.

## 5. Conclusion

MPCI alleviated DPN-related functional and pathological abnormalities and partially improved metabolic disturbances in HFD/STZ-induced T2DM rats. These effects were associated with remodeling of diabetes-related splenic immune transcriptional programs and attenuation of NF-κB/c-Jun–associated inflammatory signaling in sciatic nerve tissue. Serum exposure profiling showed the systemic presence of multiple MPCI-related prototype constituents and metabolites following oral administration. Overall, these exploratory findings suggest that MPCI may have potential immunomodulatory effects in T2DM-associated DPN and provide preliminary evidence for associations between splenic immune transcriptomic remodeling and peripheral nerve inflammatory signaling.

## Supporting information

S1 Fig GSEA of GO biological processes comparing Control vs. Model groups. (TIFF)

S2 Fig GSEA of GO biological processes comparing Model vs. MPCI-M groups. (TIFF)

S1 Table Primer sequences used for quantitative real-time PCR. (XLSX)

S2 Table Differentially expressed genes between Control and Model groups in spleen tissue identified by RNA sequencing. (XLSX)

S3 Table Differentially expressed genes between MPCI-treated and Model groups in spleen tissue identified by RNA sequencing. (XLSX)

S4 Table TPM-normalized expression values of selected immune- and inflammation-related genes across Control, Model, and MPCI-treated groups. (XLSX)

S5 Table UPLC–Q-TOF-MS chromatograms and identified chemical constituents of MPCI extract. (XLSX)

S6 Table Prototype constituents detected in rat plasma after oral administration of MPCI by UPLC–Q-TOF-MS. (XLSX)

S7 Table Metabolites detected in rat plasma after oral administration of MPCI by UPLC–Q-TOF-MS. (XLSX)

S8 Table Summarized raw data supporting the main findings of this study. (XLSX)

## References

[1] Hou G, Dong Y, Jiang Y, Zhao W, Zhou L, Cao S, et al. Immune inflammation and metabolic interactions in the pathogenesis of diabetic nephropathy. Front Endocrinol (Lausanne). 2025;16:1602594. doi: 10.3389/fendo.2025.1602594 40698245 PMC12279506

[2] Cheng Y, Chen Y, Li K, Liu S, Pang C, Gao L, et al. How inflammation dictates diabetic peripheral neuropathy: an enlightening review. CNS Neurosci Ther. 2024; 30(4):e14477. 10.1111/cns.14477 PMC11017439 37795833

[3] Moussa F, Taleb S, Borbjerg MK, Croosu SS, Mørch CD, Frøkjær JB, et al. Corneal immune cells and their relation to diabetic peripheral neuropathy and neuropathic pain. Muscle Nerve. 2026;73(1):72–8. doi: 10.1002/mus.70061 41246983

[4] Zhu J, Hu Z, Luo Y, Liu Y, Luo W, Du X, et al. Diabetic peripheral neuropathy: pathogenetic mechanisms and treatment. Front Endocrinol (Lausanne). 2024;14:1265372. doi: 10.3389/fendo.2023.1265372 38264279 PMC10803883

[5] Ashoori M, Hashemi SE, Pourahmadi M, Dadgoo M, Hosseini MS, Lotfi H, et al. Adding tibial nerve neurodynamic techniques to a rehabilitative pain management strategy improved neuropathy severity and quality of life in patients with diabetic peripheral neuropathy: a randomized sham-controlled trial. BMC Complement Med Ther. 2025;25(1):429. doi: 10.1186/s12906-025-05168-3 41286909 PMC12642109

[6] Shang Y, Pan Y, Xie L, Zhao Y, Mao W, Chen T. Neuro-immune axis in atherosclerosis: mechanisms of regulation and therapeutic opportunities. Front Immunol. 2025;16:1619338. doi: 10.3389/fimmu.2025.1619338 40977707 PMC12446013

[7] Delrue C, Speeckaert MM. Beyond blood pressure: emerging pathways and precision approaches in hypertension-induced kidney damage. Int J Mol Sci. 2025;26(15):7606. doi: 10.3390/ijms26157606 40806733 PMC12347043

[8] Gao Y, Shen S, Wang Y, Tian M. Organ crosstalk: the role of spleen. Phenomics. 2025; 5(2):192–207. 40606563 10.1007/s43657-023-00147-5 PMC12209125

[9] Hu T, Liu C-H, Lei M, Zeng Q, Li L, Tang H, et al. Metabolic regulation of the immune system in health and diseases: mechanisms and interventions. Signal Transduct Target Ther. 2024;9(1):268. doi: 10.1038/s41392-024-01954-6 39379377 PMC11461632

[10] Zhang Y, Su F, Zhu E, Sun Y, Kuang H, Wang Q. A systematical review on traditional Chinese medicine treating chronic diseases via regulating ferroptosis from the perspective of experimental evidence and clinical application. Chin Herb Med. 2025;17(2):246–60. doi: 10.1016/j.chmed.2025.01.003 40256717 PMC12009076

[11] Feng Y, Ren Y, Zhang X, Yang S, Jiao Q, Li Q, et al. Metabolites of traditional Chinese medicine targeting PI3K/AKT signaling pathway for hypoglycemic effect in type 2 diabetes. Front Pharmacol. 2024;15:1373711. doi: 10.3389/fphar.2024.1373711 38799166 PMC11116707

[12] Zhang L, Guo S, Gao Y, Jiang M, Liu Q, Yang W, et al. Effects of tangduqing granules on insulin resistance and their association with AhR and PPARs regulation in Type 2 diabetic rats exposed to persistent organic pollutants. Nat Prod Commun. 2024;19(12):1934578X241310007.

[13] Mizuno S, Kurobe-Takashima Y, Kuriki D, Susaki K, Otsuka K, Tsuchihashi T, et al. Phosphatidylcholine suppresses inflammatory responses in LPS-stimulated MG6 microglial cells by inhibiting NF-κB/JNK/p38 MAPK signaling. PLoS One. 2025;20(7):e0328206. doi: 10.1371/journal.pone.0328206 40720378 PMC12303320

[14] de Molon RS, Vernal R, Oliveira GE, Steffens JP, Ervolino E, Theodoro LH, et al. Inflammatory bone loss and signaling pathways in periodontitis: mechanistic insights and emerging therapeutic strategies. Bone Res. 2026;14(1):1. doi: 10.1038/s41413-025-00478-1 41484074 PMC12764867

[15] Lalaoui N, Masters SL. Monogenic disorders of the TNF signalling pathway. Nat Rev Rheumatol. 2026;22(1):8–25. doi: 10.1038/s41584-025-01327-5 41381726

[16] Hoffmann A, Cheng G, Baltimore D. NF-κB: master regulator of cellular responses in health and disease. Immun Inflamm. 2025;1(1):2. doi: 10.1007/s44466-025-00014-0 41669117 PMC12883533

[17] Zhang T, Zhang D, Zhang Z, Tian J, An J, Zhang W, et al. Alpha-lipoic acid activates AMPK to protect against oxidative stress and apoptosis in rats with diabetic peripheral neuropathy. Hormones (Athens). 2023;22(1):95–105. doi: 10.1007/s42000-022-00413-7 36289188

[18] Dobin A, Davis CA, Schlesinger F, Drenkow J, Zaleski C, Jha S, et al. STAR: ultrafast universal RNA-seq aligner. Bioinformatics. 2013;29(1):15–21. doi: 10.1093/bioinformatics/bts635 23104886 PMC3530905

[19] Liao Y, Smyth GK, Shi W. featureCounts: an efficient general purpose program for assigning sequence reads to genomic features. Bioinformatics. 2014;30(7):923–30. doi: 10.1093/bioinformatics/btt656 24227677

[20] Love MI, Huber W, Anders S. Moderated estimation of fold change and dispersion for RNA-seq data with DESeq2. Genome Biol. 2014;15(12):550. doi: 10.1186/s13059-014-0550-8 25516281 PMC4302049

[21] Subramanian A, Tamayo P, Mootha VK, Mukherjee S, Ebert BL, Gillette MA, et al. Gene set enrichment analysis: a knowledge-based approach for interpreting genome-wide expression profiles. Proc Natl Acad Sci U S A. 2005;102(43):15545–50. doi: 10.1073/pnas.0506580102 16199517 PMC1239896

[22] Yao X, Wang X, Zhang R, Kong L, Fan C, Qian Y. Dysregulated mast cell activation induced by diabetic milieu exacerbates the progression of diabetic peripheral neuropathy in mice. Nat Commun. 2025;16(1):4170. doi: 10.1038/s41467-025-59562-z 40325050 PMC12052842

[23] Nashtahosseini Z, Eslami M, Paraandavaji E, Haraj A, Dowlat BF, Hosseinzadeh E, et al. Cytokine signaling in diabetic neuropathy: a key player in peripheral nerve damage. Biomedicines. 2025;13(3):589. doi: 10.3390/biomedicines13030589 40149566 PMC11940495

[24] Pérez Hernández MF, Rodríguez Guerrero E, Ocharan Hernández ME, Gómez Jiménez DC, Gómez Esquivel ML, Aguilar Castillo S de J, et al. Effect of electroacupuncture for pain relief in diabetic distal symmetric polyneuropathy: a pilot randomized clinical trial. BMC Complement Med Ther. 2025;25(1):438. doi: 10.1186/s12906-025-05135-y 41366387 PMC12690971

[25] Panou T, Gouveri E, Popovic DS, Papazoglou D, Papanas N. The role of inflammation in the pathogenesis of diabetic peripheral neuropathy: new lessons from experimental studies and clinical implications. Diabetes Ther. 2025;16(3):371–411. doi: 10.1007/s13300-025-01699-7 39928224 PMC11868477

[26] Yang Y, Zhao B, Wang Y, Lan H, Liu X, Hu Y, et al. Diabetic neuropathy: cutting-edge research and future directions. Signal Transduct Target Ther. 2025;10(1):132. doi: 10.1038/s41392-025-02175-1 40274830 PMC12022100

[27] Ziegler D, Porta M, Papanas N, Mota M, Jermendy G, Beltramo E, et al. The role of biofactors in diabetic microvascular complications. Curr Diabetes Rev. 2022;18(4):e250821195830. doi: 10.2174/1871527320666210825112240 34433401 PMC10155884

[28] Baker RG, Hayden MS, Ghosh S. NF-κB, inflammation, and metabolic disease. Cell Metab. 2011;13(1):11–22. doi: 10.1016/j.cmet.2010.12.008 21195345 PMC3040418

[29] Singh A, Shadangi S, Gupta PK, Rana S. Type 2 diabetes mellitus: a comprehensive review of pathophysiology, comorbidities, and emerging therapies. Compr Physiol. 2025;15(1):e70003. doi: 10.1002/cph4.70003 39980164

[30] Tian H, An L, Wang P, Zhang X, Gao W, Li X. Review of Astragalus membranaceus polysaccharides: extraction process, structural features, bioactivities and applications. Chin Herb Med. 2024;17(1):56–69. doi: 10.1016/j.chmed.2024.09.004 39949812 PMC11814244

[31] Skovsø S. Modeling type 2 diabetes in rats using high fat diet and streptozotocin. J Diabetes Investig. 2014;5(4):349–58. doi: 10.1111/jdi.12235 25411593 PMC4210077

[32] Gheibi S, Kashfi K, Ghasemi A. A practical guide for induction of type-2 diabetes in rat: Incorporating a high-fat diet and streptozotocin. Biomed Pharmacother. 2017;95:605–13. doi: 10.1016/j.biopha.2017.08.098 28881291

[33] Zhang M, Lv X-Y, Li J, Xu Z-G, Chen L. The characterization of high-fat diet and multiple low-dose streptozotocin induced type 2 diabetes rat model. Exp Diabetes Res. 2008;2008:704045. doi: 10.1155/2008/704045 19132099 PMC2613511

[34] Barrière DA, Noll C, Roussy G, Lizotte F, Kessai A, Kirby K, et al. Combination of high-fat/high-fructose diet and low-dose streptozotocin to model long-term type-2 diabetes complications. Sci Rep. 2018;8(1):424. doi: 10.1038/s41598-017-18896-5 29323186 PMC5765114

[35] Lewis SM, Williams A, Eisenbarth SC. Structure and function of the immune system in the spleen. Sci Immunol. 2019;4(33):eaau6085. doi: 10.1126/sciimmunol.aau6085 30824527 PMC6495537

[36] Yang C, Xue Q, Feng Y, Ding W, Lu Y, Wang Q. High-dimensional phenotyping reveals novel macrophage-like and hybrid subsets within murine splenic conventional dendritic cells. PLoS One. 2026;21(1):e0341819. doi: 10.1371/journal.pone.0341819 41615933 PMC12857976

[37] Lim H, Lee H, Noh K, Lee SJ. IKK/NF-κB-dependent satellite glia activation induces spinal cord microglia activation and neuropathic pain after nerve injury. Pain. 2017;158(9):1666–77. doi: 10.1097/j.pain.0000000000000959 28722693

[38] Abdelkader NF, Ibrahim SM, Moustafa PE, Elbaset MA. Inosine mitigated diabetic peripheral neuropathy via modulating GLO1/AGEs/RAGE/NF-κB/Nrf2 and TGF-β/PKC/TRPV1 signaling pathways. Biomed Pharmacother. 2022;145:112395. doi: 10.1016/j.biopha.2021.112395 34775239

[39] Yao H, Feng J, Zheng Q, Wei Y, Yang G, Feng W. Comparison of the effects of prophylactic and therapeutic administrations on peripheral neuropathy in streptozotocin-diabetic rats with gliclazide or methylcobalamin. Exp Clin Endocrinol Diabetes. 2020;128(10):635–43. doi: 10.1055/a-0635-0672 30453342

[40] Li Z, Wang W, Meng F, Zhou Z, Zhao Z, Mei Z. Analgesic and neuroprotective effects of Baimai Ointment on diabetic peripheral neuropathy. J Ethnopharmacol. 2022;292:115122. doi: 10.1016/j.jep.2022.115122 35202714

[41] Le TT, Jung Y, Cha JW, Tran SH, Bang M, Kang TK. Morutamins A-L from Morus alba L. root bark with inhibitory effects on epidermal growth factor receptor and toll-like receptor-mediated NF-kappaB/AP‑1 activation. ACS Omega. 2026;11(1):2246–62. 41552606 10.1021/acsomega.5c11810 PMC12809818

[42] He S, Liang X, Chen W, Nima Y, Li Y, Gu Z, et al. Osthole ameliorates chronic pruritus in 2,4-dichloronitrobenzene-induced atopic dermatitis by inhibiting IL-31 production. Chin Herb Med. 2024;17(2):368–79. doi: 10.1016/j.chmed.2024.01.003 40256714 PMC12009079

[43] El-Sherbiny GM, Kalaba MH. Marine carotenoids: a critical review of bioactivities, bioavailability, and therapeutic potential. Biomed Res Int. 2025;2025:4147524. doi: 10.1155/bmri/4147524 41473736 PMC12746009

