# Supplementary figures and images for: Immunomodulatory effects of a multi-component pharmacological intervention on diabetic peripheral neuropathy in type 2 diabetic rats: An exploratory study
Source: PLoS One. 2026 Jun 4;21(6):e0350984. doi: 10.1371/journal.pone.0350984 (PMC13235885; doi:10.1371/journal.pone.0350984)

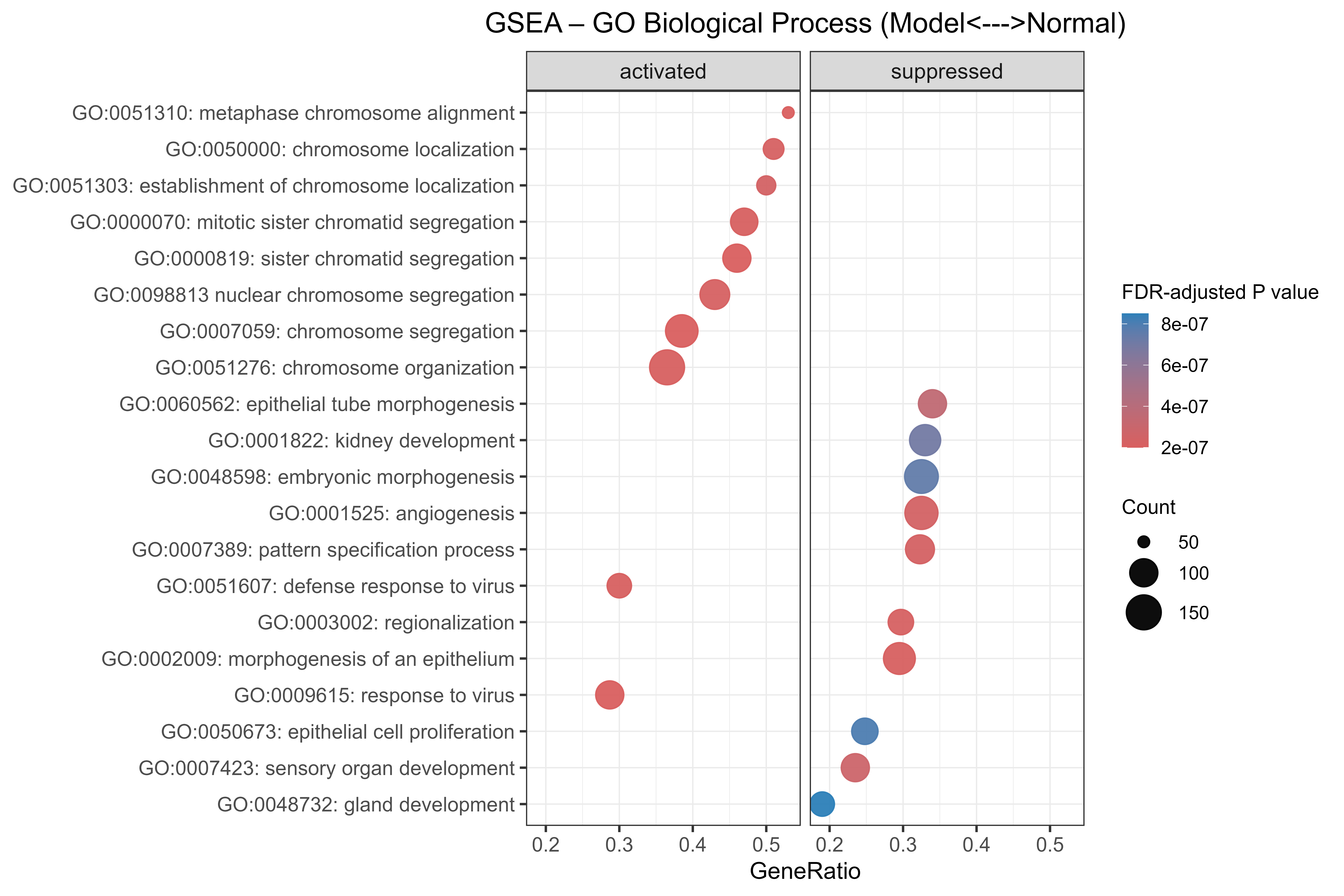

Supplement: S1 Fig — (TIFF) [file pone.0350984.s001.tiff]

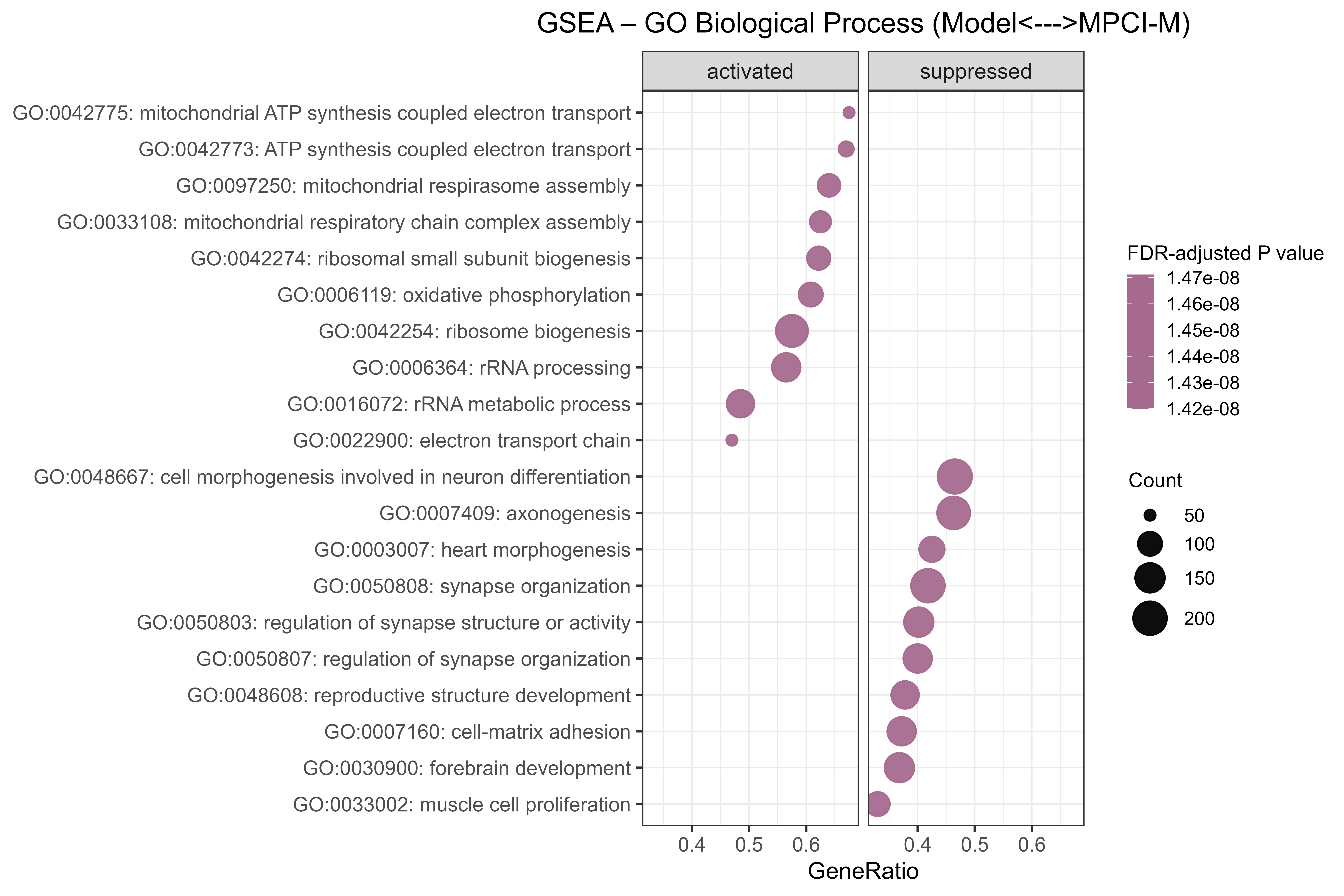

Supplement: S2 Fig — (TIFF) [file pone.0350984.s002.tiff]
